# The association between history of diabetic foot ulcer, perceived health and psychological distress: the Nord-Trøndelag Health Study

**DOI:** 10.1186/1472-6823-9-18

**Published:** 2009-08-25

**Authors:** Marjolein M Iversen, Kristian Midthjell, Grethe S Tell, Torbjørn Moum, Truls Østbye, Monica W Nortvedt, Sverre Uhlving, Berit R Hanestad

**Affiliations:** 1 Faculty of Health and Social Sciences, Bergen University College, PO Box 7030, 5020 Bergen, Norway; 2 Department of Public Health and Primary Health Care, University of Bergen, P.O. Box 7804, 5020 Bergen, Norway; 3 The HUNT Research Center, Norwegian University of Science and Technology, Neptunveien 1, 7650 Verdal, Norway; 4 Department of Behavioural Sciences in Medicine, University of Oslo, 0317 Oslo, Norway; 5 Department of Community and Family Medicine, Duke University Medical Center, Box 104006 DUMC, Durham, North Carolina 27710, USA; 6 Department of Internal Medicine, Stavanger University Hospital, Box 8100, 4068 Stavanger, Norway

## Abstract

**Background:**

While the adverse impact of a history of a foot ulcer on physical health among persons with diabetes is well known, little is known about the association between foot ulcer, perceived health and psychological distress. Results from various studies are difficult to compare as different study designs, samples and/or different questionnaires have been used. The aim of this study was to compare levels of anxiety and depression, psychological well-being and perceived health between persons with diabetes, with or without a history of foot ulcer, and persons without diabetes in a large study of community-dwelling individuals.

**Methods:**

This study included 65,126 persons, of whom 63,632 did not have diabetes, 1,339 had diabetes without a history of foot ulcer and 155 had diabetes and a history of foot ulcer. Levels of anxiety and depression were assessed by the Hospital Anxiety and Depression Scale (HADS). Psychological well-being was measured on a four-item scale, and perceived health was measured with a one-item question. We investigated whether levels of anxiety, depression, psychological well-being and perceived health were different in the three study groups using multiple regression models controlling for demographic factors, body mass index, smoking and cardiovascular conditions. Separate multivariate analyses comparing the two diabetes samples were additionally adjusted for diabetes-specific variables.

**Results:**

A history of foot ulcer was significantly associated with more depressive symptoms, poorer psychological well-being and poorer perceived health compared to participants without diabetes. In multivariate analyses, perceived health and psychological well-being were significantly poorer among those with a history of foot ulcer compared to those without diabetes. Among persons with diabetes, perceived health was significantly worse among those with a history of foot ulcer. After multivariate adjustment, levels of anxiety and depression and psychological well-being did not differ between the two diabetes groups.

**Conclusion:**

Perceived health and psychological well-being were significantly poorer among participants with diabetes and a history of foot ulcer compared to those without diabetes. Among people with diabetes, a history of foot ulcer had significant negative impact on perceived health but did not independently contribute to psychological distress.

## Background

Foot ulceration is a common and disabling complication of diabetes, and the lifetime risk of a person with diabetes developing this complication may be as high as 25% [1]. A history of previous diabetic foot ulceration increases the risk for new ulceration. Foot ulcers precede approximately 85% of all diabetic lower extremity amputations, and the mortality following amputation is high [2].

The possibility of negative psychological effects of diabetic foot ulceration, beyond that of diabetes in itself, has been highlighted [3-5]. Even among people with their first diabetic foot ulcer, one-third suffer from clinical depression [6]. In addition, a prospective study found that healing of a foot ulcer did not lead to improved mental or general health (measured by SF-36) [7]. Similarly, Goodridge [8] and Meyer [9] did not find differences in mental health between those with healed versus unhealed diabetic foot ulcers. On the other hand, Ragnarson-Tennvall [4] reported that those with healed ulcers had less anxiety and depression and better self-reported health than those with current ulcers. Interpretation of these results is hampered by small sample size [8,9], lack of non-diabetic comparison groups or diabetic groups without foot ulcer history [4,7,8], and lack of adjustment for important demographic [8] and lifestyle variables [4,8]. In addition, some studies did not control for the impact of cardiovascular disease or other complications when assessing the effect of a history of foot ulcer on mental health [7,8].

In order to overcome some of these shortcomings, the aim of this study was to compare symptom levels of anxiety and depression, as well as psychological well-being and perceived health, between i) persons with diabetes who reported a history of foot ulcer, ii) persons with diabetes without a history of foot ulcer, and iii) persons without diabetes. If differences were found, we wanted to further examine whether these could be explained by demographic characteristics, lifestyle factors and cardiovascular disease status, in addition to diabetes-specific variables such as duration of diabetes, insulin use and long-term glucose control (HbA_1c_). The Nord-Trøndelag Health Study (HUNT2) afforded the investigation of these aims in a very large population-based sample of men and women. Participants with self-reported diabetes were well characterized with regard to their diabetes illness and perceived health, as well as several aspects of psychological distress such as symptoms of anxiety, depression and psychological well-being. In addition, measures of demographic and lifestyle factors were included.

## Methods

### Settings

The second round of the population-based Nord-Trøndelag Health Study (HUNT2) was carried out from 1995 to 1997. The source population is stable and ethnically homogeneous (3% are of non-Caucasian origin). The design and methods have been described elsewhere [10].

### Study population

Of the total number invited, 65,604 individuals (71%) aged 20 years or more attended HUNT2 and answered a short questionnaire (Q1) before participating in a brief health examination/screening. In addition, all participants received a second questionnaire (Q2) with a prestamped, addressed envelope. A total of 1,972 respondents answered affirmatively to the question, "Do you have, or have you had, diabetes?" (Q1) and were invited to take part in the diabetes substudy. This involved an additional questionnaire (Q3) on diabetes-related issues, including diagnosis, treatment, duration and complications, including a history of foot ulcer. A total of 1,692 persons (85.8%) with diabetes returned this questionnaire. Out of 1,494 responses to the question "Have you had a foot ulcer that required more than three weeks to heal?", 155 persons answered affirmatively, comprising the subgroup with diabetes and a history of foot ulcer. The remaining 1,339 participants comprised the subgroup of those reporting diabetes without a history of foot ulcer [11]. Some 63,632 participants reported not having diabetes.

### Diabetes classification

In HUNT2, a non-fasting serum sample was analysed for glucose; for those who reported diabetes, an EDTA whole blood sample was also analysed for HbA_1c_. Those who reported diabetes were given a follow-up appointment (74.8% participation) where a fasting blood sample was drawn and analysed for glucose, C-peptide and GAD antibodies, allowing the differentiation between type 1 and type 2 diabetes.

### Questionnaires

Questionnaires were used to assess symptom levels of anxiety and depression, psychological well-being and perceived health. Anxiety and depression were assessed by the Hospital Anxiety and Depression Scale (HADS) [12]. This instrument consists of 14 items, of which seven measure anxiety (HADS-A subscale) and seven measure depression (HADS-D subscale). Missing substitution was performed for individuals who had answered five or six of the seven HADS-A or HADS-D questions. This was done by multiplying the obtained score by 7/5 if five of the seven questions were answered and by 7/6 if six questions were answered. Such missing substitution was done for 12.1% of participants for the HADS-A scale and 5.8% for HADS-D scale. Some 6.2% and 4.6% answered fewer than five questions on HADS-A and HADS-D, respectively, and were excluded. Each item is scored from 0 to 3; thus, the maximum score is 21 on each of the subscales. Higher scores indicate higher levels of symptom load. Caseness is usually defined by a score of 8 or above on HADS-D or HADS-A. This cut-off level has been shown to balance sensitivity and specificity optimally on receiver operating characteristic (ROC) curves [12]. To enhance the specificity of depression disorders and anxiety, a cut-off point of 11 has also been used [13]. Previously, factor analyses of HADS in HUNT have been shown to result in a two-factor solution consistent with the two subscales of anxiety and depression [14]. Regarding internal consistency, Cronbach's alpha for the anxiety and depression subscales in HUNT has been found to be 0.80 and 0.76, respectively [14].

Psychological well-being was self-assessed by four questions related to various aspects of psychological well-being such as life satisfaction, vigour, calmness and cheerfulness, and a psychological well-being index was constructed using a sum score of these four items. Because of the different response categories (ranging from four to seven), all items were transformed into scales (0–10) to provide equal weighting for each item. When one of the four items was missing, it was substituted with the mean value of the remaining three (*n *= 1,506); if two or more items were missing, the case was excluded (*n *= 11,547). A psychological well-being index was constructed using the mean of these four items; a higher score indicates a higher level of well-being. The index comprises a cognitive component (i.e., life satisfaction), as well as positive and negative affect, and thus conforms to generally accepted operationalizations of global psychological well-being [15]. The internal consistency of the psychological well-being index was high (Cronbach's alpha = 0.81), with inter-item correlations ranging from 0.47 to 0.60. The original index was first formulated in 1990 as a brief assessment of psychological distress in a broad population sample. It showed a high correlation (r = 0.85) with the more extensive Hopkins Symptom Checklist (HSCL-25) [16], and has since been used in several analyses based on the two first waves of the Nord-Trøndelag Health Study as well as in other Norwegian community-based studies [17].

Perceived health was measured by the question: "How is your health these days?" (measured on a scale from 1 = poor to 4 = very good). This single-item measure of perceived health, or a similar version, has been used in several studies, and has been shown to have acceptable psychometric properties [18].

Demographic variables were also included in the questionnaire as were questions on history of stroke, myocardial infarction and angina pectoris. The diabetes subgroups additionally indicated whether they had undergone peripheral vascular surgery or had problems with their eyes due to diabetes.

### Statistical analyses

Bivariate comparisons of means and univariate multiple linear regression analyses were used. The four dependent variables: symptom levels of anxiety (HADS-A) and depression (HADS-D), psychological well-being and perceived health were transformed to z-scores (i.e., variables with a mean of zero and a standard deviation of one) in order to facilitate comparisons of effects (mean differences) between subgroups across outcomes.

The three participant subgroups were used as an independent categorical variable, entered in the regression analyses as two dummies with the non-diabetic subgroup as reference. Other independent variables included age, gender, education, BMI (weight (kg)/height^2 ^(metres) and smoking (current versus former and non-smoker). Cardiovascular comorbidity was defined as a history of stroke, myocardial infarction or angina pectoris.

In separate univariate multiple regression analyses restricted to the two diabetes subgroups, those without a history of foot ulcer were used as the reference group. Analyses were adjusted for diabetes-specific variables (insulin use, HbA_1c _and diabetes duration), cardiovascular comorbidity and eye problems due to diabetes. Those who did not answer the question on insulin use but answered "yes" to the use of oral antidiabetic agents were classified as non-insulin users. Participants who answered "yes" to any of the questions related to history of stroke, myocardial infarction, angina pectoris or peripheral surgery were categorized as having cardiovascular comorbidity.

In separate models, the presence of effect modification (statistical interaction) was tested by adding to the full regression model multiplicative terms involving the sample subgroup variable, as well as each of the other independent variables, one pair of variables at a time. Statistical analyses were conducted using SPSS version 15 (SPSS, Chicago Il). The statistical significance level was defined as *P *< 0.05.

The HUNT2 study was approved by the Norwegian Data Inspectorate and the Regional Committee for Medical Research Ethics. Participation was voluntary, and each participant signed a written consent form. The study complied with the Declaration of Helsinki.

## Results

### Description of study groups

Compared to the non-diabetic sample, those with a history of diabetic foot ulcer were older and had higher BMI and mean waist circumference; a higher proportion were male, physically inactive, had low education, angina pectoris, myocardial infarction and stroke, and a lower proportion were smokers (see Additional file 1). Comparing the two diabetes groups, those with a history of diabetic foot ulcer had a higher mean waist circumference and level of HbA_1c_; a larger proportion were physical inactive, used insulin, had longer diabetes duration and a history of stroke, peripheral vascular surgery and eye problems due to diabetes.

Participants with a history of diabetic foot ulcer reported significantly poorer perceived health and psychological well-being compared to diabetic persons without a history of foot ulcer and to those without diabetes. The mean depression score was significantly higher in persons with a history of foot ulcer compared to non-diabetic persons (4.7 versus 3.5). Proportions with scores 8 and above were 18.8% for those with a history of foot ulcer and 10.8% for the non-diabetic group (*P *= 0.002). Percentages with scores 11 and above were 7.6% for those with a history of foot ulcer and 3.2% for the non-diabetic group (*P *= 0.002). Level of anxiety did not differ between the three subgroups.

### Predictors of psychological distress and perceived health among all study groups

Participants with diabetes with or without a history of foot ulcer had significantly higher HADS depression scores, poorer psychological well-being and worse perceived health compared to participants without diabetes (see Additional file 2). After adjustment for demographic variables, lifestyle factors and cardiovascular conditions, the findings persisted for psychological well-being and perceived health, while the association with depression scores was no longer statistically significant. In the final multivariate model, older age, female gender, low education, high BMI, current smoking and a history of stroke and angina pectoris were significantly associated with poorer psychological well-being and perceived health. A history of foot ulcer was more strongly related to perceived health than to psychological well-being (see Additional file 2).

For anxiety, depression, and perceived health, there were interactions with age, showing that persons below 55 years with diabetes consistently had the poorest outcome (*P *values for interaction terms were 0.002, 0.045, < 0.001, respectively). In the non-diabetic group, higher education was associated with better perceived health, whereas among persons with diabetes and a history of foot ulcer, higher education was associated with poor perceived health. Furthermore, the negative impact of angina pectoris and stroke on perceived health was stronger in the non-diabetic population sample than among persons with diabetes.

### Predictors of psychological distress and perceived health among diabetes subgroups

When comparing the two diabetes groups, a history of foot ulcer was significantly associated with poorer perceived health, while no differences were found for levels of anxiety, depression or psychological well-being (see Additional file 3). The association between worse perceived health and a history of diabetic foot ulcer persisted after adjustment for demographic variables, lifestyle factors, cardiovascular conditions and the diabetes specific variables. In the final multivariate model, older age, higher BMI, eye problems due to diabetes and cardiovascular comorbidity were also significant; however, diabetes-specific variables such as insulin use, HbA_1c _and diabetes duration were not.

## Discussion

In this large population-based study, perceived health and psychological well-being were significantly poorer among those with diabetes and a history of foot ulcer than among those without diabetes. Comparing the diabetes groups, perceived health was significantly worse among those with a history of foot ulcer, while no differences between the groups were found for levels of anxiety, depression or psychological well-being.

HUNT2 is to our knowledge the largest, non-selected population-based study of diabetes-related foot ulcers, including over 60,000 participants. Three outcome measures – anxiety and depression (HADS), psychological well-being and perceived health – allow for a broad view of the studied field. Although self-reported diabetes was validated by blood tests, it is nevertheless likely that some subjects with diabetes were included in the non-diabetic group. Among those without known diabetes, a total of 62,757 delivered a non-fasting venous blood sample for glucose measurement. Of these, 217 persons had glucose levels above 11 mmol/l, and this group was followed up separately, but not included in the group with known diabetes due to uncertainty as to whether this was a permanent condition. Therefore it might be that the number of subjects classified as having diabetes based on self-report is underestimated. Although history of foot ulcer in people with diabetes is self-reported and has inherent limitations, it was not feasible to clinically validate the diagnosis in this large epidemiological study. Even though some participants may erroneously have reported other types of ulcers, such as venous leg ulcers, the term *foot ulcer *(*fotsår*) is probably less ambiguous in Norwegian than in English. Not including data on neuropathy and nephropathy in the models could be seen as a limitation. However measurement of neuropathy and nephropathy was not feasible in this large epidemiological study.

Both diabetes groups reported worse perceived health than the non-diabetic group. This is in accordance with results from other studies indicating that perceived health is affected by chronic illness such as diabetes, and people with diabetes typically rate their health worse than non-diabetic people [19]. Perceived health is thought to reflect the underlying disease burden [20] and has been shown to be a good predictor of mortality [18]. In our study, perceived health was significantly worse among those with a history of diabetic foot ulcer than among those without. The association between a foot ulcer and health has also been shown in a previous study among people with current diabetic foot ulcers [5]. In that study Ribu and collaborators showed that those with current foot ulcers have poorer health status than diabetic patients without foot ulcers and the general population. Another study found that those with primary healed ulcers had better perceived health than those with current ulcers [4]. Our results indicated that a history of foot ulcer had an independent impact on perceived health over and above the underlying diabetes itself.

Previous studies [21,22] have found that there may not be enough focus on the prevention of foot ulcers in diabetic persons. By assessing perceived health, health care professionals may identify vulnerable patients with diabetes and might offer these patients more individual support and an appropriate foot care program. Future studies should examine whether perceived health is a predictor of excess mortality in patients with a history of foot ulcer.

The two diabetic samples reported poorer psychological well-being than the non-diabetic sample. Although the presence of diabetes-related complications has been reported to be associated with psychological distress [23,24], we are not aware of published studies including participants with a history of foot ulcer and a non-diabetic comparison group. Studies using focus group interviews have found that people with a current foot ulcer report emotions of frustration, anger and guilt about the possible development of new ulcers and threat of amputations [25]. Results from our study indicate that such feelings may persist after the ulcer has healed, as our measure of psychological well-being incorporates such aspects as life satisfaction, vigour, calmness and cheerfulness. In HUNT2, a history of foot ulcer, stroke and angina pectoris had similar associations with psychological well-being and perceived health, indicating that the burden of a history of foot ulcer is comparable to the perceived burden of stroke and angina pectoris.

Our findings of higher rates of depression in people with diabetes than in non-diabetic participants are in agreement with results from previous studies [26,27]. Ismail [6] has shown that up to one-third of people with their first diabetic foot ulcer suffer from clinical depression. In our study, the proportion with a history of foot ulcer and symptoms of depression (HADS-D ≥ 8) was lower (18.8%). One possible reason may be that our question was about a history of foot ulcer and not a current foot ulcer. Among subjects with diabetes, those who also have complications are more likely to have depressive disorders than those who do not [28]. This was confirmed for complications such as cardiovascular comorbidity or eye problems due to diabetes, which had an independent impact on depression. The lack of an independent association between diabetic foot ulcers and depression was in line with Vileikyte et al [29]. Others, however, have reported that depressive symptoms are associated with impaired healing and recurrence of ulcers in elderly type 2 diabetic patients [30]. In addition, increasing evidence points to the importance of assessing diabetes-specific and/or ulcer-specific distress rather than just generalized distress [31,32]. Neuropathy and its symptoms, including pain, loss of feeling, and especially unsteadiness, seem to be particularly important determinants of depression [29,33].

Diabetes-specific variables such as insulin use, diabetes duration and the level of HbA_1c _were not significantly associated with perceived health or psychological distress in the multivariate analyses among diabetic persons with or without a history of foot ulcer. This is in accordance with the study of Ismail [6] who found no association between depression and glycaemic control among people with their first foot ulcer. In previous studies among persons with diabetes, obesity was associated with a higher likelihood of depression [34]. This was confirmed in the present study, where obesity had an independent association with depression, poorer psychological well-being and poorer perceived health. Thus, health care personnel should pay attention to the possibility of psychological distress in obese diabetic patients.

## Conclusion

We found the impact of a history of foot ulcer on perceived health to be distinct from the association of the underlying diabetes. Furthermore, a history of foot ulcer, stroke and angina pectoris had similar associations with perceived health and psychological well-being. Thus, there are several possible predictors of poorer perceived health and psychological well-being, including a history of foot ulcer. The clinical picture in patients who have had diabetes for a long time may be complex, with several complications appearing at the same time, perhaps affecting their ability to self-manage their diabetes. Focus on perceived health may help to identify vulnerable patients with diabetes and to offer these patients more intensive individual support and a foot care program.

In summary, perceived health and psychological well-being were significantly poorer among participants with diabetes and a history of foot ulcer than among those without diabetes. Among people with diabetes, a history of foot ulcer had a significant impact on perceived health but did not have an independent effect on psychological distress.

## Competing interests

The authors declare that they have no competing interests.

## Authors' contributions

All authors (MMI, BRH, GST, KM, TØ, TM, MWN, SU) participated in the design of the study and helped to draft or revise the manuscript. KM and SU participated in the acquisition of data. MMI and TM performed the statistical analyses. All authors have read and approved the final manuscript.

## Pre-publication history

The pre-publication history for this paper can be accessed here:



## Supplementary Material

Additional file 1 **Table 1**. Description of the study population: the HUNT2 study. ^a ^Sample sizes vary somewhat depending on the actual completion of the different tests/questionnaires. ^b ^Significance of *t *test or χ^2 ^test for difference between subjects with a history of diabetic foot ulcers and those without diabetes. ^c ^Significance of *t *test or χ^2 ^test for difference between subjects with and without a history of diabetic foot ulcer. ^d ^*P *value reflects test of current smokers vs. never + former smokers combined. Click here for file

Additional file 2 **Table 2**. Predictors of HADS-anxiety, HADS-depression, psychological well-being and perceived health in the three study groups. The three subgroups are: non-diabetic subjects, diabetic subjects with and without a history of foot ulcer. All dependent variables have been transformed to z-scores. Unstandardized regression coefficients. ^a ^Higher scores on HADS-anxiety or -depression reflect more symptoms of anxiety or depression. ^b ^Higher scores of psychological well-being or perceived health reflect better psychological well-being or better perceived health. ^c ^Only individuals with responses on all independent variables were included in the bivariate analyses. ^d ^Multivariate analyses with all variables in the table included. ^e ^*P *< 0.001. ^f ^*P *< 0.01. Click here for file

Additional file 3 **Table 3**. Predictors of HADS-anxiety, HADS-depression, psychological well-being and perceived health among diabetic persons with and without a history of foot ulcer. All dependent variables have been transformed to z-scores. Unstandardized regression coefficients. ^a ^Higher scores on HADS-anxiety or -depression reflect more symptoms of anxiety or depression. ^b ^Higher scores of psychological well-being or perceived health reflect better psychological well-being or better perceived health. ^c ^Only individuals with responses on all independent variables were included in the bivariate analyses. ^d ^Multivariate analyses with all variables in the table included. ^e ^*P *< 0.001. ^f ^*P *< 0.01. ^g ^*P *< 0.05. Click here for file
