# Supplementary material for: The association between history of diabetic foot ulcer, perceived health and psychological distress: the Nord-Trøndelag Health Study
Source: BMC Endocr Disord. 2009 Aug 25;9:18. doi: 10.1186/1472-6823-9-18 (PMC2737541; doi:10.1186/1472-6823-9-18)
Supplement: Additional file 1 — Table 1. Description of the study population: the HUNT2 study. a Sample sizes vary somewhat depending on the actual completion of the different tests/questionnaires. b Significance of t test or χ2 test for difference between subjects with a history of diabetic foot ulcers and those without diabetes. c Significance of t test or χ2 test for difference between subjects with and without a history of diabetic foot ulcer. d P value reflects test of current smokers vs. never + former smokers combined. [file 1472-6823-9-18-S1.doc]

Table 1: Description of the study population: the HUNT2 study

| Characteristics | Non-diabetic subjects  n = 63,632^a^ | Diabetic subjects  without a history of foot ulcer  n = 1,339^a^ | Diabetic subjects  with a history of foot ulcer  n = 155^a^ | *P* value^b^ | *P* value^c^ |
| --- | --- | --- | --- | --- | --- |
| Demographic characteristics |  |  |  |  |  |
| Age (years) | 49.7 (SD 17.3) | 65.6 (SD 13.6) | 67.2 (SD 14.0) | < 0.001 | 0.157 |
| Male sex (%) | 46.7 | 49.7 | 56.8 | 0.012 | 0.097 |
| Single (%) | 40.1 | 38.1 | 45.8 | 0.150 | 0.064 |
| Education (≥ 10 years) (%) | 64.0 | 37.7 | 33.8 | < 0.001 | 0.367 |
| Lifestyle characteristics |  |  |  |  |  |
| BMI (kg/m^2^) | 26.3 (SD 4.1) | 28.9 (SD 4.8) | 29.3 (SD 5.3) | < 0.001 | 0.396 |
| Waist circumference (cm) | 86.2 (SD 11.6) | 95.0 (SD 12.0) | 98.2 (SD 12.3) | < 0.001 | 0.002 |
| Physical activity < 1 hr/week (%) | 19.8 | 27.5 | 37.2 | < 0.001 | 0.026 |
| Current smokers (%) | 29.0 | 16.8 | 11.1 | < 0.001^d^ | 0.070^d^ |
| Former smokers (%) | 24.9 | 33.5 | 34.0 |  |  |
| Never smokers (%) | 46.0 | 49.7 | 54.9 |  |  |
| Cardiovascular disease status |  |  |  |  |  |
| Self-reported stroke (%) | 1.8 | 5.0 | 12.2 | < 0.001 | < 0.001 |
| Self-reported myocardial infarction (%) | 3.0 | 12.6 | 15.3 | < 0.001 | 0.345 |
| Self-reported angina pectoris (%) | 4.6 | 18.5 | 22.0 | < 0.001 | 0.307 |
| Subgroups of diabetes |  |  |  |  |  |
| Type 1 (%) |  | 16.9 | 26.0 |  |  |
| Type 2 (%) |  | 83.1 | 74.0 |  |  |
| Diabetes-specific variables |  |  |  |  |  |
| HbA_1c_ (% units) | – | 8.1 (SD 1.7) | 8.4 (SD 2.0) | – | 0.015 |
| Insulin use (%) | – | 31.8 | 43.5 | – | 0.004 |
| Duration of diabetes (years) (median) | – | 6.0 | 10.0 | – | 0.001 |
| Peripheral vascular surgery (%) | – | 2.7 | 10.7 | – | < 0.001 |
| Eye problems due to diabetes (%) | – | 11.9 | 24.8 | – | < 0.001 |
| Psychological assessment |  |  |  |  |  |
| Perceived health score (1–4) | 2.9 (SD 0.7) | 2.4 (SD 0.6) | 2.3 (SD 0.6) | < 0.001 | 0.002 |
| Psychological well-being score (0–10) | 7.0 (SD 1.6) | 6.7 (SD 1.6) | 6.4 (SD 1.6) | < 0.001 | 0.017 |
| HADS-anxiety score (0–21) | 4.3 (SD 3.3) | 4.1 (SD 3.5) | 4.0 (SD 3.8) | 0.482 | 0.920 |
| HADS-depression score (0–21) | 3.5 (SD 3.1) | 4.3 (SD 3.4) | 4.7 (SD 3.6) | < 0.001 | 0.180 |
| HADS-anxiety (score ≥ 8) (%) | 15.5 | 15.7 | 18.9 | 0.273 | 0.335 |
| HADS-depression (score ≥ 8) (%) | 10.8 | 17.1 | 18.8 | 0.002 | 0.614 |
| HADS-anxiety (score ≥ 11) (%) | 5.3 | 6.4 | 8.3 | 0.116 | 0.398 |
| HADS-depression (score ≥ 11) (%) | 3.2 | 6.0 | 7.6 | 0.002 | 0.439 |

^a^ Sample sizes vary somewhat depending on the actual completion of the different tests/questionnaires.

^b^ Significance of *t* test or *χ^2^* test for difference between subjects with a history of diabetic foot ulcers and those without diabetes.

^c^ Significance of *t* test or *χ^2^* test for difference between subjects with and without a history of diabetic foot ulcer.

^d^ *P* value reflects test of current smokers vs. never + former smokers combined.
