# Supplementary material for: The association between history of diabetic foot ulcer, perceived health and psychological distress: the Nord-Trøndelag Health Study
Source: BMC Endocr Disord. 2009 Aug 25;9:18. doi: 10.1186/1472-6823-9-18 (PMC2737541; doi:10.1186/1472-6823-9-18)
Supplement: Additional file 2 — Table 2. Predictors of HADS-anxiety, HADS-depression, psychological well-being and perceived health in the three study groups. The three subgroups are: non-diabetic subjects, diabetic subjects with and without a history of foot ulcer. All dependent variables have been transformed to z-scores. Unstandardized regression coefficients. a Higher scores on HADS-anxiety or -depression reflect more symptoms of anxiety or depression. b Higher scores of psychological well-being or perceived health reflect better psychological well-being or better perceived health. c Only individuals with responses on all independent variables were included in the bivariate analyses. d Multivariate analyses with all variables in the table included. e P < 0.001. f P < 0.01. [file 1472-6823-9-18-S2.doc]

Table 2: Predictors of HADS-anxiety, HADS-depression, psychological well-being and perceived health in the three study groups

|  | HADS-anxiety^a^  (*n* = 58,513) | | HADS-depression^a^  (*n* = 59,283) | | Psychological well-being^b^  (*n* = 50,905) | | Perceived health^b^  (*n* = 60,224) | |
| --- | --- | --- | --- | --- | --- | --- | --- | --- |
|  | Bivariate^c^ | Multivariate^d^ | Bivariate^c^ | Multivariate^d^ | Bivariate^c^ | Multivariate^d^ | Bivariate^c^ | Multivariate^d^ |
| Subgroups |  |  |  |  |  |  |  |  |
| No diabetes | Ref. | Ref. | Ref. | Ref. | Ref. | Ref. | Ref. | Ref |
| No history of diabetic foot ulcer | –0.051 | 0.013 | 0.278^e^ | 0.034 | –0.182^e^ | –0.114^e^ | –0.654^e^ | –0.246^e^ |
| A history of diabetic foot ulcer | –0.073 | 0.012 | 0.355^e^ | 0.072 | –0.348^e^ | –0.279^f^ | –0.943^e^ | –0.479^e^ |
| Demographic variables |  |  |  |  |  |  |  |  |
| Age (in decades) | –0.034^e^ | –0.051^e^ | 0.135^e^ | 0.105^e^ | –0.036^e^ | –0.015^e^ | –0.210^e^ | –0.150^e^ |
| Male gender | –0.197^e^ | –0.186^e^ | 0.079^e^ | 0.084^e^ | 0.118^e^ | 0.120^e^ | 0.069^e^ | 0.077^e^ |
| Education ≥ 10yr | –0.071^e^ | –0.123^e^ | –0.341^e^ | –0.142^e^ | 0.156^e^ | 0.085^e^ | 0.542^e^ | 0.198^e^ |
| Lifestyle variables |  |  |  |  |  |  |  |  |
| BMI (kg/m^2^) | –0.006^e^ | –0.002 | 0.021^e^ | 0.011^e^ | –0.006^e^ | –0.005^e^ | –0.038^e^ | –0.023^e^ |
| Smoking (yes/no) | 0.189^e^ | 0.166^e^ | 0.113^e^ | 0.154^e^ | –0.194^e^ | –0.205^e^ | –0.142^e^ | –0.220^e^ |
| Cardiovascular co-morbidity |  |  |  |  |  |  |  |  |
| Stroke (yes/no) | 0.060 | 0.125^e^ | 0.582^e^ | 0.304^e^ | –0.369^e^ | –0.281^e^ | –0.902^e^ | –0.428^e^ |
| Angina pectoris (yes/no) | 0.037 | 0.158^e^ | 0.429^e^ | 0.119^e^ | –0.276^e^ | –0.217^e^ | –0.974^e^ | –0.484^e^ |
| Myocardial infarction (yes/no) | –0.081^f^ | –0.029 | 0.366^e^ | 0.001 | –0.163^e^ | –0.015 | –0.802^e^ | –0.157^e^ |
| R^2^_adj_. |  | 0.023 |  | 0.065 |  | 0.021 |  | 0.171 |

The three subgroups are: non-diabetic subjects, diabetic subjects with and without a history of foot ulcer. All dependent variables have been transformed to *z* scores. Unstandardized regression coefficients.

^a^ Higher scores on HADS-anxiety or -depression reflect more symptoms of anxiety or depression.

^b^ Higher scores of psychological well-being or perceived health reflect better psychological well-being or better perceived health.

^c^ Only individuals with responses on all independent variables were included in the bivariate analyses

^d^ Multivariate analyses with all variables in the table included.

^e^ *P* < 0.001

^f^ *P* < 0.01
