# Supplementary material for: The association between history of diabetic foot ulcer, perceived health and psychological distress: the Nord-Trøndelag Health Study
Source: BMC Endocr Disord. 2009 Aug 25;9:18. doi: 10.1186/1472-6823-9-18 (PMC2737541; doi:10.1186/1472-6823-9-18)
Supplement: Additional file 3 — Table 3. Predictors of HADS-anxiety, HADS-depression, psychological well-being and perceived health among diabetic persons with and without a history of foot ulcer. All dependent variables have been transformed to z-scores. Unstandardized regression coefficients. a Higher scores on HADS-anxiety or -depression reflect more symptoms of anxiety or depression. b Higher scores of psychological well-being or perceived health reflect better psychological well-being or better perceived health. c Only individuals with responses on all independent variables were included in the bivariate analyses. d Multivariate analyses with all variables in the table included. e P < 0.001. f P < 0.01. g P < 0.05. [file 1472-6823-9-18-S3.doc]

Table 3: Predictors of HADS-anxiety, HADS-depression, psychological well-being and perceived health among diabetic persons with and without a history of foot ulcer

|  | HADS-anxiety^a^  (*n* = 1,085) | | HADS-depression^a^  (*n* = 1,118) | | Psychological well-being^b^  (*n* = 1,083) | | Perceived health^b^  (*n* = 1,148) | |
| --- | --- | --- | --- | --- | --- | --- | --- | --- |
|  | Bivariate^c^ | Multivariate^d^ | Bivariate^c^ | Multivariate^d^ | Bivariate^c^ | Multivariate^d^ | Bivariate^c^ | Multivariate^d^ |
| Diabetes subgroups |  |  |  |  |  |  |  |  |
| No history of diabetic foot ulcer | Ref. | Ref. | Ref. | Ref. | Ref. | Ref. | Ref. | Ref. |
| A history of diabetic foot ulcer | 0.014 | 0.008 | 0.102 | 0.021 | –0.198 | –0.149 | –0.315^e^ | –0.220^g^ |
| Demographic variables |  |  |  |  |  |  |  |  |
| Age (in decades) | –0.079^e^ | –0.131^e^ | 0.084^e^ | 0.051 | –0.008 | 0.022 | –0.147^e^ | –0.105^e^ |
| Male gender | –0.302^e^ | –0.282^e^ | –0.039 | 0.036 | 0.155^g^ | 0.135^g^ | 0.164^f^ | 0.093 |
| Education ≥ 10yr | –0.143^g^ | –0.189^f^ | –0.236^e^ | –0.138 | 0.129^g^ | 0.071 | 0.275^e^ | 0.053 |
| Lifestyle variables |  |  |  |  |  |  |  |  |
| BMI (kg/m^2^) | 0.013 | 0.006 | 0.019^f^ | 0.019^f^ | – 0.019^f^ | –0.017^g^ | –0.029^e^ | –0.026^e^ |
| Current smoking (yes/no) | 0.159 | 0.154 | 0.058 | 0.109 | –0.160^g^ | –0.198^g^ | 0.067 | –0.048 |
| Diabetes-specific variables |  |  |  |  |  |  |  |  |
| Insulin use (yes/no) | –0.007 | –0.092 | –0.023 | –0.015 | –0.014 | 0.028 | 0.043 | –0.009 |
| HbA_1c_ (%) | –0.035 | –0.035 | 0.000 | –0.022 | 0.005 | 0.020 | –0.025 | –0.006 |
| Duration of diabetes (yrs) | 0.002 | 0.004 | 0.006 | 0.006 | –0.005 | –0.005 | –0.002 | –0.001 |
| Co-morbidity |  |  |  |  |  |  |  |  |
| Eye problems due to diabetes (yes/no) | 0.195^g^ | 0.171 | 0.294^f^ | 0.226^g^ | –0.261^f^ | –0.188^g^ | –0.332^e^ | –0.201^g^ |
| Cardiovascular co-morbidity (yes/no) | 0.049 | 0.159^g^ | 0.247^e^ | 0.172^g^ | –0.215^f^ | –0.226^f^ | –0.440^e^ | –0.317^e^ |
| R^2^_adj_. |  | 0.048 |  | 0.028 |  | 0.029 |  | 0.102 |

All dependent variables have been transformed to z-scores. Unstandardized regression coefficients.

^a^ Higher scores on HADS-anxiety or -depression reflect more symptoms of anxiety or depression.

^b^ Higher scores of psychological well-being or perceived health reflect better psychological well-being or better perceived health.

^c^ Only individuals with responses on all independent variables were included in the bivariate analyses

^d^ Multivariate analyses with all variables in the table included.

^e^ *P* < 0.001

^f^ *P* < 0.01

^g^ *P* < 0.05
